# Plumericin Modulates the AhR–NFκB–Nrf2 Signaling Network to Counteract Indoxyl Sulfate-Induced Intestinal Epithelial Cells Impairment

**DOI:** 10.3390/ijms27010293

**Published:** 2025-12-27

**Authors:** Rosaria Margherita Rispoli, Stefan Schwaiger, Ada Popolo, Giuseppina Autore, Marco Gargaro, Hermann Stuppner, Stefania Marzocco

**Affiliations:** 1 Department of Pharmacy, University of Salerno, Via Giovanni Paolo II 132, 84084 Fisciano, Italy; 2 PhD Program in Drug Discovery and Development, University of Salerno, Via Giovanni Paolo II 132, 84084 Fisciano, Italy; 3 Institute of Pharmacy/Pharmacognosy and Center for Molecular Biosciences Innsbruck (CMBI), University of Innsbruck, 6020 Innsbruck, Austria; 4 Department of Medicine and Surgery, University of Perugia, 06132 Perugia, Italy

**Keywords:** indoxyl sulfate, plumericin, chronic kidney disease, apoptosis, inflammation, oxidative stress, wound repair

## Abstract

Intestinal impairment plays a pivotal role in many chronic conditions, including chronic kidney disease (CKD), a progressive disorder affecting over 800 million people worldwide. CKD does not only affect the kidney, but it is recognized as a systemic condition characterized by chronic low-grade inflammation, that contributes to disease progression and associated complications. The intestine is one of the major sources of CKD-associated inflammation, also due to the production and accumulation of some uremic toxins, normally excreted by healthy kidneys, such as indoxyl sulfate (IS). IS is a pro-inflammatory and pro-oxidant protein-bound uremic toxin that increases intestinal epithelial permeability, promotes microbial translocation, and enhances inflammatory and oxidative responses. Although IS-induced intestinal damage has been documented, the underlying molecular mechanisms and effective therapeutic strategies to counteract its effects remain to be elucidated. Against this backdrop in the present study, we investigated the impact of plumericin, an iridoid spironolactone, on IS-induced intestinal impairment using IEC-6, an intestinal epithelial cells model. In IS-treated IEC-6, plumericin reduces apoptosis, inhibits inflammation and oxidative stress, and restores epithelial wound repair. In these conditions plumericin also promotes Nrf-2 and inhibits NF-kB and AhR activation induced by IS. Moreover, the same inhibitory effect of plumericin on inflammation and oxidative stress and in promoting wound repair is also observed in the presence of IS and pro-inflammatory stimuli, as occurs in CKD considering the associated systemic low-grade inflammation. These findings suggest that plumericin may represent a promising therapeutic candidate for intestinal impairment in CKD acting with an integrated mechanism.

## 1. Introduction

Alterations in intestinal homeostasis are being increasingly recognized as a common feature of many chronic diseases. Disruptions in this balance can lead to systemic inflammation and contribute to the progression of various comorbidities. In this context, maintaining intestinal homeostasis has a crucial role in systemic health, as it regulates the interaction between the gut microbiota and host tissues [1]. In the context of chronic kidney disease (CKD), this balance is disrupted, contributing to a chronic low-grade inflammatory state and the progression of systemic complications, including cardiovascular, immune, neurological, and gastrointestinal disorders [2,3,4]. In fact, the intestine is increasingly recognized as a key site of inflammatory and immune dysregulation in CKD, where both the epithelial and the resident immune cells actively respond to the accumulation of uremic toxins by triggering inflammatory and oxidative stress pathways. Uremic toxins originating from cellular metabolism are classified as water-soluble and low molecular weight compounds with different protein-bound affinity. Among them, some uremic toxins, including indoxyl sulfate (IS) are produced by gut bacteria and are not removed by current conventional dialysis [5,6]. In fact, about 97% of the IS pool in the body is protein-bound and its accumulation in CKD can reach 80 times the concentrations observed in healthy individuals, encompassing concentration ranges from 40 to 494 µmol/L [7,8,9,10]. IS compromises intestinal barrier homeostasis by disrupting tight junctions and promoting epithelial damage [11,12]. The subsequent increase in intestinal permeability allows endotoxins and bacterial components, such as lipopolysaccharide (LPS), to translocate from the intestinal lumen to the lamina propria and reach the systemic circulation [13]. Moreover, IS has been reported to induce oxidative stress, increasing the generation of reactive oxygen species (ROS) through the activation of NADPH oxidases (NOX). This thereby promotes the activation of NF-κB and the consequent upregulation of inflammasome component expression, including NLRP3 and pro-caspase-1. These events lead to the activation of caspase-1 and the maturation and secretion of IL-1β, which further amplifies the inflammatory process [14]. The pro-inflammatory effect induced by IS at the intestinal level is also mediated by the induction of inducible nitric oxide synthase (iNOS) and cyclooxygenase-2 (COX-2), which contribute to the production of pro-inflammatory cytokines, including IL-6 and TNF-α. This further compromises epithelial homeostasis and promotes intestinal damage. Furthermore, IS inhibits the nuclear factor (erythroid-derived 2)-like 2 (Nrf2)/Keap1 pathway, thereby reducing the transcription of antioxidant enzymes such as HO-1, NQO1, and SOD in intestinal epithelial cells [15,16]. Collectively, these effects contribute to the “leaky gut” phenotype commonly observed in patients with CKD, establishing IS as a critical mediator linking intestinal dysfunction, systemic inflammation, and oxidative stress in the progression of CKD [17]. In the light of this, there has been a growing interest in compounds that can reduce inflammation and restore redox balance while preserving the integrity of the epithelial barrier, particularly natural compounds [18,19,20]. Our interest focused on plumericin, an iridoid lactone isolated from *Himatanthus sucuuba.* Preparations of the stem bark and latex of the Amazonian tree *H. sucuuba* (Spruce ex Müll.Arg.) Woodson (Apocynaceae) have been traditionally used in South America as anti-inflammatory, antitumor, analgesic, and antiulcer agents [21]. The spirolactone iridoid plumericin, a major bioactive constituent of *H. sucuuba*, has been shown to exhibit antiparasitic, anti-microbial, and antifungal activities [22,23,24]. Previous studies showed that plumericin potently inhibits NF-κB pathway [25] and TNF-induced senescence of endothelial cells [26,27]. Moreover, we reported that plumericin inhibits apoptosis and inflammatory and oxidative stress response while increasing wound repair both in vitro and in vivo [28,29]. This was carried out using a model of lipopolysaccharide from *E.coli* plus interferon gamma induced inflammation in intestinal cells and a model of chemically induced colitis by dinitrobenzene sulfonic acid (DNBS), widely used to study inflammatory bowel disease such as Chron’s disease [28,29]. In light of this previous evidence, in this study we evaluated the potential of plumericin for a different condition of intestinal impairment and inflammation specifically due to IS, an aryl hydrocarbon receptor activator. Thus, we had a different target with respect to the previously evaluated model of intestinal impairment for plumericin, with a potential interest addressed to CKD associated intestinal impairment.

## 2. Results

### 2.1. Plumericin Reduced Apoptosis Induced by IS in IEC-6 Cells

**Figure 1:**
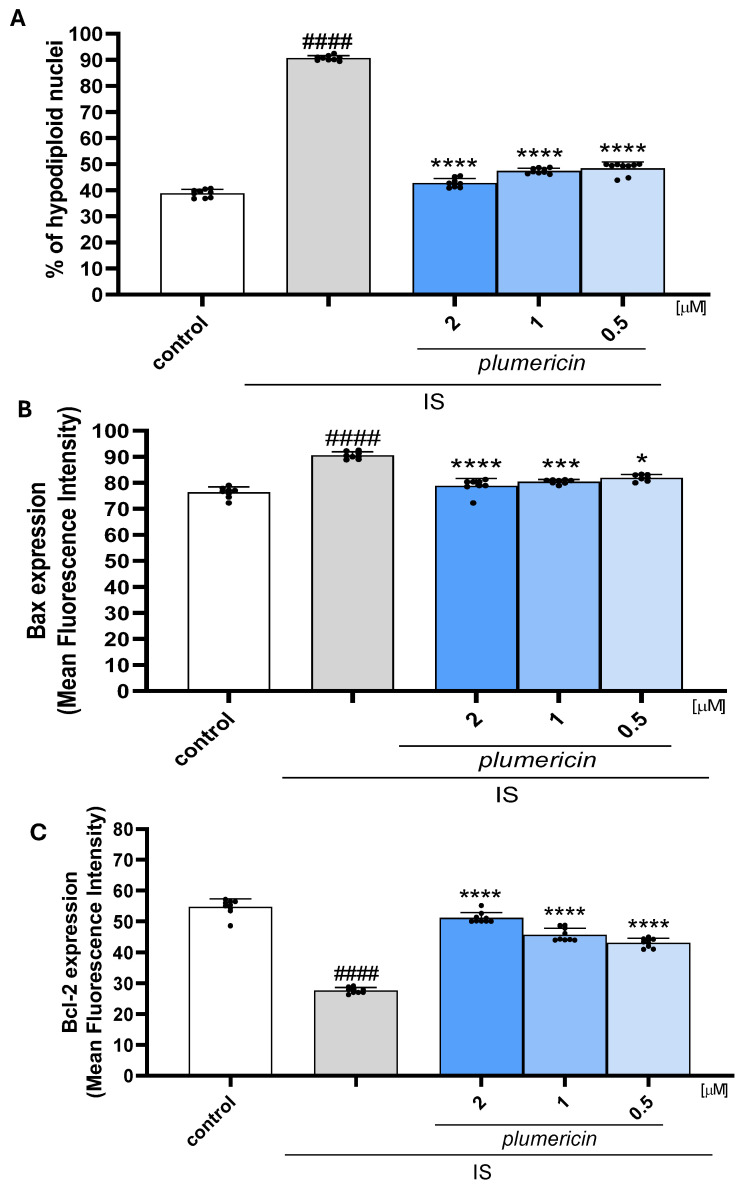
Effect of plumericin on apoptosis (**A**), Bax (**B**), and Bcl-2 (**C**) expression evaluated by cytofluorimetry. Data are reported as percentage of hypodipoloid nuclei ((**A**); *n* = 9) or as mean fluorescence intensity ((**B**); *n* = 9 and (**C**); *n* = 9). Comparisons were performed using a one-way analysis of variance, and multiple comparisons were made by Bonferroni’s post-test. #### indicate *p* < 0.0001 vs. untreated cells (control); *, *** and **** indicate *p* < 0.05, *p* < 0.001, and *p* < 0.0001 vs. IS, respectively.

Previous studies reported the pro-apoptotic effect of IS [12,30]. In order to investigate the protective effect of plumericin on this aspect, we evaluated apoptosis by cytofluorimetric analysis of PI-stained hypodiploid nuclei. Our results indicated that plumericin (2-1-0.5 µM) significantly reduced IS-induced apoptosis in IEC-6 cells (*p* < 0.05 vs. IS; Figure 1A). To further investigate the anti-apoptotic efficacy of plumericin, we evaluated the expression levels of Bax, a pro-apoptotic protein, and Bcl-2, an anti-apoptotic proteins, by cytofluorimetric analysis. Our results showed that plumericin (2-1-0.5 μM) significantly decreased Bax expression (*p* < 0.05 vs. IS; Figure 1B) and increased Bcl-2 expression (*p* < 0.0001 vs. IS; Figure 1C) at all concentrations tested in IS IEC-6. Plumericin at the tested concentrations did not affect cell viability (Supplementary Figure S1).

### 2.2. Plumericin Inhibits iNOS and COX-2 Expression Inflammasome Activation in IS-Treated IEC-6 Cells

**Figure 2:**
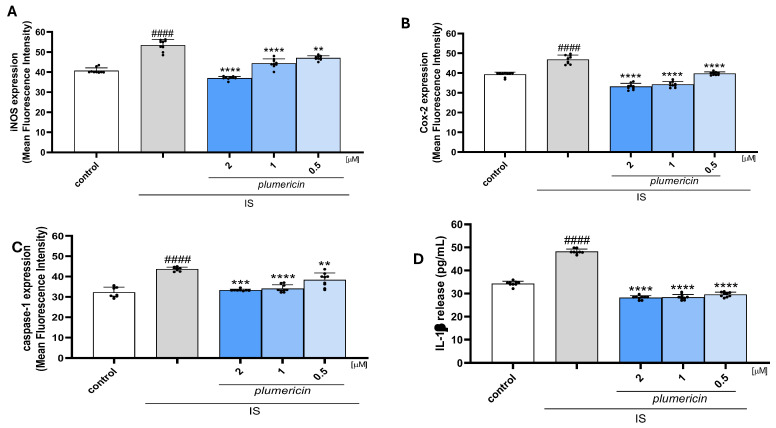
Effect of plumericin on iNOS, COX-2 expression and inflammasome activation in IEC-6 cells. Plumericin significantly inhibited iNOS (**A**), COX-2 (**B**), and caspase-1 (**C**) expression, evaluated by cytofluorimetry, and IL-1β release (**D**), evaluated by an enzyme-linked immuno sorbent assay. Data are reported as mean fluorescence intensity ((**A**–**C**); *n* = 9) or as pg/mL of IL-1β release ((**D**); *n* = 9). Comparisons were performed using a one-way analysis of variance, and multiple comparisons were made by Bonferroni’s post-test. #### indicate *p* < 0.0001 vs. untreated cells (control); **, *** and **** indicate *p* < 0.01, *p* < 0.001, and *p* < 0.0001 vs. IS respectively.

The effect of plumericin on iNOS and COX-2 expression in IS treated IEC-6 cells was evaluated using cytofluorimetric analysis. The findings of this study demonstrated that plumericin (2-1-0.5 µM) significantly inhibited the expression of both iNOS (*p* < 0.01 vs. IS; Figure 2A) and COX-2 (*p* < 0.0001 vs. IS; Figure 2B) at all concentrations tested. To further assess the anti-inflammatory potential of plumericin, the inflammasome activation was also evaluated. We observed that plumericin (2-1-0.5 µM), significantly inhibited caspase-1 expression (*p* < 0.01 vs.. IS: Figure 2C) and IL-1β production (*p* < 0.0001 vs. IS; Figure 2D) in IS-stimulated IEC-6 at all concentrations tested. N(ω)-nitro-l-arginine methyl ester (L-NAME;1 μM), used as positive control, significantly inhibited iNOS expression compared to IS alone (*p* < 0.0001 vs. IS; mean = 33.330 ± 0.260) while indomethacin (1 μM), used as positive control, significantly inhibited COX-2 expression compared to IS alone (*p* < 0.0001 vs. IS; mean = 33.490 ± 0.733; Supplementary Figure S2A,B).

### 2.3. Plumericin Inhibits ROS Release, Nrf2 Nuclear Translocation and Modulate Superoxide Dismutase (SOD)-2 Expression in IS-Treated IEC-6 Cells

**Figure 3:**
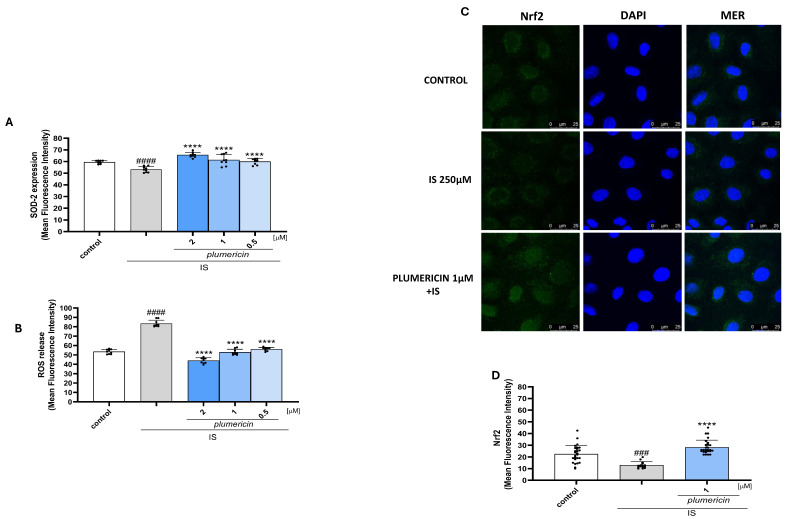
Effect of plumericin [1 μM] on on SOD-2 expression (**A**) evaluated by cytofluorimetry and ROS release (**B**). Data are reported as mean fluorescence intensity (*n* = 9). Comparisons were performed using a one-way analysis of variance and multiple comparisons were made by Bonferroni’s post-test. #### denote *p* < 0.0001 vs. untreated cells (control); ### denote *p* < 0.001 vs control; **** indicate *p* < 0.0001 vs. IS. Effect of plumericin (1 μM) on Nrf2 activation, evaluated by immunofluorescence analysis (**C**) and mean fluorescence quantification (**D**). The blue fluorescence identified the nuclei, whereas the green fluorescence indicated Nrf2. Scale bar: 25 µm.

To investigate the effect of plumericin in counteracting the IS-induced oxidative stress in IEC-6 cells, we evaluated intracellular ROS release. Our results showed that plumericin significantly inhibited ROS release in IEC-6 cells (*p* < 0.0001 vs. IS; Figure 3A). To further explore the potential antioxidant effect of plumericin, we evaluated its effect on Nrf2 nuclear translocation. Following activation, this factor translocates from the cytosol to the nucleus where it binds to specific sequences, thereby influencing the expression of downstream genes that are involved in regulating the antioxidant cellular response. In our experiments, Nrf2 was labelled with a green fluorescence probe, to track the effect of plumericin (1 µM) on its nuclear translocation. As shown in Figure 3C,D, plumericin induced Nrf2 nuclear translocation that was inhibited by treatment with IS (250 µM). Moreover, we also assessed whether plumericin (2-1-0.5 µM) modulated the expression of the antioxidant enzyme, SOD, whose expression was downregulated by IS. Our results showed that SOD expression was restored by plumericin at all concentrations tested (*p* < 0.0001 vs. IS; Figure 3B). L-glutathione reduced (GSH; 10 µM), used as positive control, significantly inhibited ROS release (*p* < 0.0001 vs. IS; mean = 41.946 ± 2.25) and increased SOD-2 expression compared to IS alone (*p* < 0.0001 vs. IS; mean = 67.797 ± 1.17; Supplementary Figure S2C,D).

### 2.4. Plumericin Enhances Wound Closure and Restores E-Cadherin and ZO-1 Expression on IS Treated IEC-6 Cells

**Figure 4:**
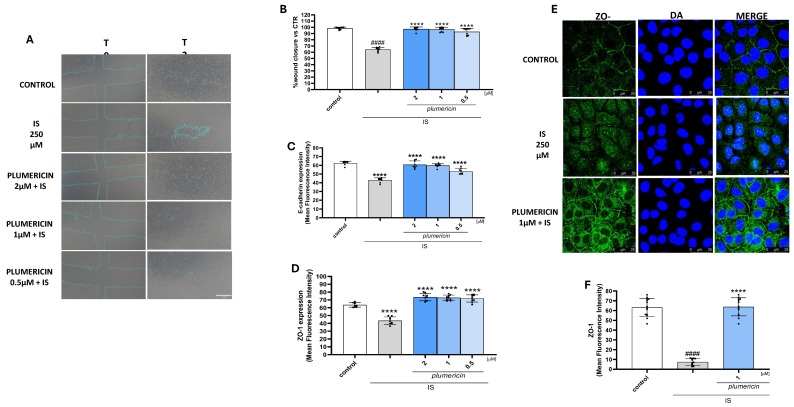
Representative pictures representing the wound repair induced by mechanical scratch in IEC-6 after 24 h (**A**) from plumericin (2-1-0.5 µM) and IS treatment (250 μM) and the quantitative analysis expressed as percentage of wound closure at 24 h with respect to time 0 ((**B**); *n* = 9). Effect of plumericin on E-cadherin expression ((**C**); *n* = 9) and ZO-1 expression evaluated by cytofluorimetric analysis ((**D**); *n* = 9). ZO-1 localization (**E**) was evaluated by immunofluorescence analysis (*n* = 3) and (**F**) reports mean fluorescence quantification. Comparisons were performed using a one-way analysis of variance and multiple comparisons were made by Bonferroni’s post-test. #### indicates *p* < 0.0001 vs. untreated cells (control); **** indicates *p* < 0.0001 vs. IS.

To assess the effect of plumericin on the reconstitution process at the intestinal level, we carried out a wound-healing assay on IS-treated IEC-6 monolayers. Once the cultured cells had reached confluence, a wound was created in IEC-6 monolayers by scraping, and a 5× phase contrast objective was used to monitor cellular migration at the wound site. Our results indicated that plumericin (2-1-0.5 µM) induced wound closure comparable to the control at all concentrations tested (*p* < 0.0001 vs. IS; Figure 4B and Supplementary Figure S3). To evaluate the markers involved in maintaining the integrity of the intestinal barrier, we performed a cytofluorimetric analysis to measure the expression of E-cadherin and ZO-1 proteins. IS downregulated the expression of these proteins (*p* < 0.0001; Figure 4C,D). Plumericin restored the expression of these proteins at all concentrations tested (*p* < 0.0001 vs. IS; Figure 4C,D). Furthermore, immunofluorescence analysis showed that IS altered ZO-1 localization, while plumericin [1 µM] restored it (Figure 4E).

### 2.5. Plumericin Inhibits NF-κB p65 Translocation in IS-Treated IEC-6 Cells

**Figure 5:**
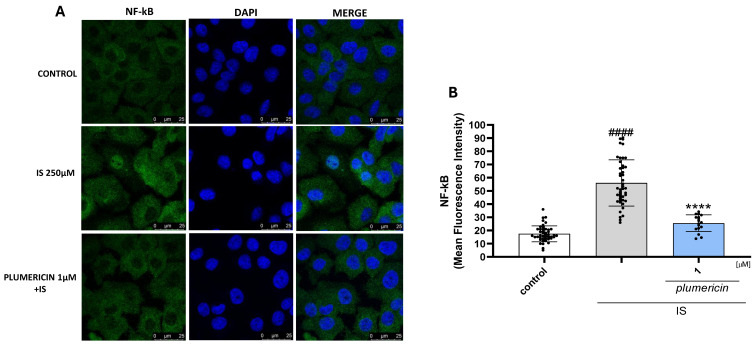
Effect of plumericin (1 μM) on NF-kB p65 translocation, evaluated by immunofluorescence analysis (**A**) and mean fluorescence quantification (**B**). The blue fluorescence identified the nuclei, whereas the green fluorescence indicated NF-kB p65. Scale bar: 25 µm. #### denote *p* < 0.0001 vs control; **** indicates *p* < 0.0001 vs. IS.

After p65 subunit phosphorylation, the free NF-κB dimers translocate into the nucleus and bind to specific sequences to regulate downstream gene expression. Thus, we labelled p65 with a green fluorescent antibody to track the influence of plumericin (1 µM), added 1 h before IS (250 µM) on NF-κB translocation. As shown in Figure 5, nuclear p65 levels increased after 1 h of IS treatment. NF-κB translocation was found to be further reduced by plumericin in intestinal cells when compared to IS alone.

### 2.6. Plumericin Inhibits IS-Induced AhR Expression in IEC-6 Cells

**Figure 6:**
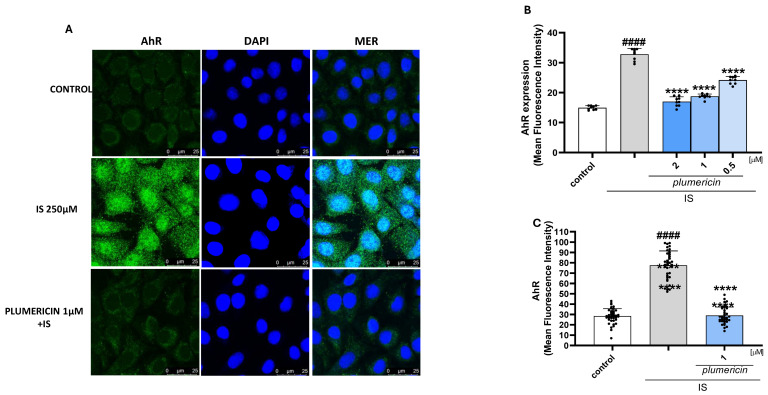
Effect of plumericin on AhR nuclear translocation (**A**), evaluated by immunofluorescence analysis and expression evaluated by cytofluorimetric analysis (**B**). In panel B, data are reported as mean fluorescence intensity (*n* = 9) while in panel C data are reported as mean fluorescence quantification (**C**). Comparisons were performed using a one-way analysis of variance and multiple comparisons were made by Bonferroni’s post-test. #### indicates *p* < 0.0001 vs. untreated cells (control); **** indicates *p* < 0.0001 vs. IS.

The aryl hydrocarbon receptor (AhR) was identified as the binding partner of IS [31]. To further investigate the effect of plumericin on IS activated cellular pathway, we evaluated its nuclear translocation, by immunofluorescence analysis, and expression, evaluated by cytofluorimetric analysis. Our data demonstrated that plumericin was able to reduce both the nuclear translocation of the receptor (Figure 6A) and its expression (*p* < 0.0001 vs. IS; Figure 5B) induced by IS (*p* < 0.0001 vs. control).

### 2.7. Plumericin Counteracts IS Inflammatory and Oxidative Responses and Preserves Epithelial Integrity in IEC-6 Cells in Inflammatory Conditions

**Figure 7:**
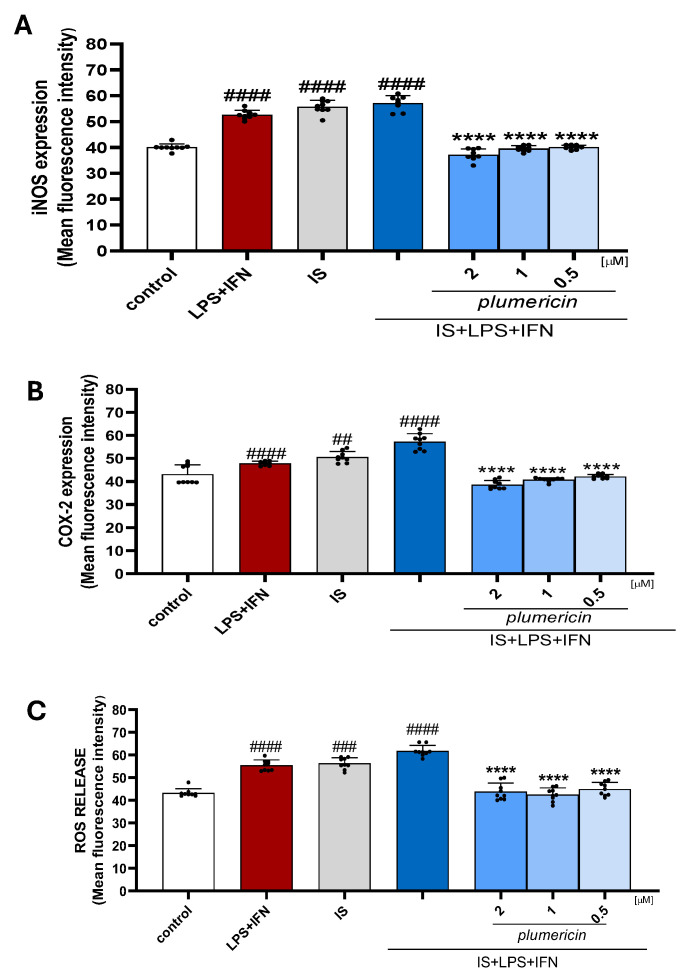
Effect of plumericin on iNOS (**A**), COX-2 expression (**B**), and ROS release (**C**) induced by IS + LPS + IFN evaluated by cytofluorimetric analysis. Data are reported as mean fluorescence intensity (*n* = 9). Comparisons were performed using a one-way analysis of variance and multiple comparisons were made by Bonferroni’s post-test. ##, ### and #### indicate *p* > 0.01, *p* < 0.001 and *p* < 0.0001 vs. untreated cells (control), respectively; **** indicates *p* < 0.0001 vs. IS + LPS + IFN.

**Figure 8:**
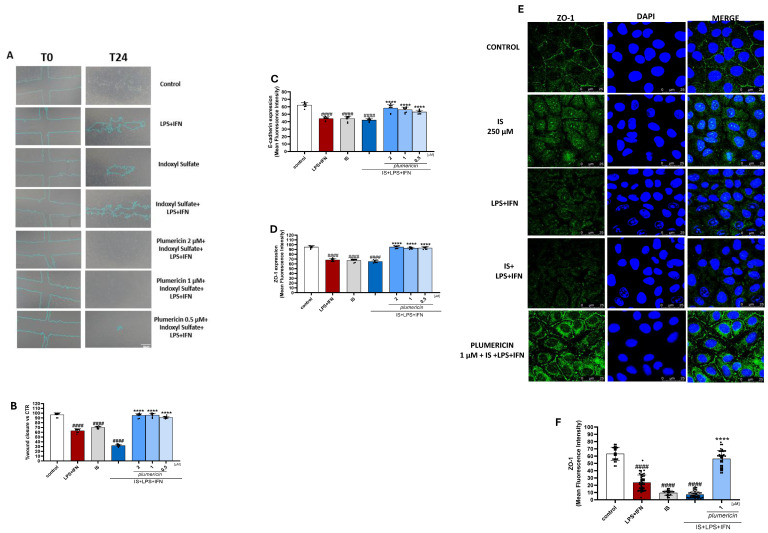
Pictures representing the wound repair induced by mechanical scratch in IEC-6 after 24 h (**A**) from plumericin (2-1-0.5 µM) and IS + LPS + IFN treatment (Scale bar: 50 µm), and the quantitative analysis expressed as percentage of wound closure at 24 h with respect to time 0 (**B**). Effect of plumericin on E-cadherin (**C**) and ZO-1 (**D**) expression (**E**) evaluated by cytofluorimetric analysis. Data are reported as mean fluorescence intensity (*n* = 9). Effect of plumericin (1 µM) on ZO-1 localization (**E**) evaluated by immunofluorescence analysis The blue fluorescence identified the nuclei, whereas the green fluorescence indicated ZO-1. Mean fluorescence quantification of immunofluorescence analysis is reported in panel (**F**). Scale bar: 25 µm. Comparisons were performed using a one-way analysis of variance and multiple comparisons were made by Bonferroni’s post-test. #### indicates *p* < 0.0001 vs. untreated cells (control), respectively; **** indicates *p* < 0.0001 vs. IS + LPS + IFN.

In CKD, an elevated plasma level of uremic toxins was observed, along with increased intestinal permeability. This, in turn, was shown to promote the translocation of bacterial components, such as LPS, into the systemic circulation [32]. Chronic exposure to LPS was demonstrated to contribute to the development of low-grade chronic inflammation by triggering high levels of cytokine production and the presence of mediators such as interferon-γ (IFN-γ), which were shown to strengthen the immune response. The combined effects of uremic toxins, LPS, and inflammatory cytokines create a vicious cycle of inflammation, oxidative stress, and tissue damage in the intestine and systemic circulation. In this context, we evaluated the potential of plumericin to mitigate the inflammatory state in the intestine and investigated the interaction between IS and inflammatory mediators. The results shown below indicate that plumericin can counteract the damage caused by exposure to both IS and LPS + IFN by modulating the expression of inflammatory markers such as ROS, INOS, and COX-2, while preserving the structural integrity of cells through epithelial restoration and normalization of epithelial junctions (*p* < 0.0001 vs. IS + LPS + IFN; Figure 7 and Figure 8; Supplementary Figure S4).

## 3. Discussion

Impaired intestinal function and low-grade inflammation are increasingly recognized as key factors associated with various chronic diseases, including CKD [3]. IS is the third most prevalent toxin after guanidinosuccinic acid and methylguanidine in CKD patients and is one of the most biologically active uremic toxins directly involved in the pathogenesis and progression of CKD [15,16,17,18,19,20,21,22,23,24,25,26,27,28,29,30]. In this context, there is growing interest in identifying new therapeutic strategies capable of mitigating IS effects. Natural plant-derived compounds have emerged as promising candidates, useful in treating altered intestinal homeostasis in many chronic diseases as well as in intestinal inflammation [19]. In this regard, the present study evaluated the potential effects of plumericin, an iridoid lactone isolated from *H. sucuuba*, against intestinal IS-induced alterations. In CKD many factors, among them uremic toxins like IS, can trigger inflammation which can result in cellular apoptosis [33]. In our current study, we report that IS induces activation of the apoptotic pathway in intestinal epithelial cells through downregulation of BCL-2 and upregulation of BAX, confirming existing knowledge about the pro-apoptotic effect of IS. Plumericin effectively counteracts IS-induced IEC-6 apoptosis also reducing pro apoptotic BAX and enhancing anti apoptotic BCL-2 factors. This effect of plumericin is in accordance with previous studies in models of intestinal inflammation in vitro and in vivo where it significantly reduced apoptotic parameters (including reduced BAX expression and increased BCL-2/BCL-xL) [28].

Chronic inflammation in CKD further contributes to enhance apoptosis, thereby generating a self-perpetuating cycle where elevated apoptosis leads to intestinal barrier dysfunction and increased permeability, with the exacerbation of CKD-related complication such as an inflammatory chronic state. IS induces the overexpression of pro-inflammatory mediators such as iNOS and COX-2 in several cell types, including intestinal epithelial cells [16,34,35]. Moreover, IS enhances NLRP3 inflammasome activation, thereby amplifying tissue inflammation [36,37]. In this study we confirmed the IS-induced iNOS and COX-2 over-expression and inflammasome activation in IEC-6. Plumericin inhibits all these pro-inflammatory factors induced by IS in IEC-6. This effect is particularly relevant in the context of CKD, where iNOS, COX-2, and the NLRP3 inflammasome are key mediators of uremic inflammation [38,39]. In fact, IS promotes the sustained induction of iNOS, resulting in the excessive formation of nitric oxide and peroxynitrite, that drive oxidative stress and endothelial dysfunction [15]. Meanwhile, COX-2 over-expression increases the production of pro-inflammatory prostaglandins, thereby contributing to disease progression. Furthermore, NLRP3 inflammasome activation (with increased caspase-1 activity and IL-1β maturation) amplifies the inflammatory response [40].

Oxidative stress together with high concentrations of uremic toxins can further trigger inflammation in patients with CKD [41]. In fact, several reports suggested that IS-induced ROS production results in the upregulation of inflammatory gene expression through ROS-dependent ERK/MAPK activation [42,43]. In IEC-6 cells, we previously reported that IS induces oxidative stress also through a reduction of Nrf-2 activation and its related products, such as HO-1, NQO-1, and SOD-2 [28]. IS down-regulates Nrf-2 and its downstream enzymes, reducing the epithelial antioxidant capacity and leading to mitochondrial dysfunction and increased cellular susceptibility to injury [44]. Plumericin significantly reduces IS-induced oxidative stress by inhibiting ROS release and increasing the Nrf-2 antioxidant pathway, thus suggesting the restoration of redox balance. This effect is particularly relevant because Nrf-2 and its downstream enzymes, including SOD-2, represent the primary cellular defense against oxidative damage.

Oxidative and inflammatory signaling triggered by IS, in addition to apoptosis, further impairs epithelial junctions with a reduction of wound healing capacity, ultimately compromising monolayer integrity [45]. The loss of Nrf-2-driven antioxidants, such as SOD-2, exacerbates oxidative and inflammatory signaling, further compromising epithelial integrity, disrupting tight junctions, and reducing wound-healing capacity [15]. In fact, reactivating the Nrf-2 pathway and restoring SOD-2 and related enzymes contribute to counteract IS-induced redox imbalance, thereby protecting epithelial function and barrier integrity [46].

Wound repair as a pre-requisite to re-establish the mucosal epithelial barrier and intestinal homeostasis is crucial for efficient resolution of inflammation [47]. In this study, we also observed the ability of plumericin to restore epithelial integrity in the presence of IS, promoting epithelial migration following injury and increasing the expression of E-cadherin and ZO-1, whose distribution is dysregulated by IS in IEC-6 [12,45]. Plumericin, also by reducing oxidative stress, prevents damage to junctional proteins, maintaining epithelial junctional integrity and supporting wound healing repair. Therefore, by protecting these junctional proteins, plumericin directly preserves epithelial cohesion and function, which is critical in preventing further CKD progression at intestinal level. Since the NF-κB pathway is a key regulator of the inflammatory and oxidative stress response and plumericin suppresses NF-κB, in this study we investigated if this ability could also be confirmed in IS-treated intestinal cells. Our results indicated that plumericin suppresses IS-induced NF-κB activation, as evidenced by the reduced nuclear translocation of its p65 subunit, thus giving an important insight into the mechanism-based plumericin activity in mitigating IS damage, as observed in endothelial cell models and in previous in vitro studies [25]. This effect is consistent with the known molecular mechanism of plumericin, which inhibits the NF-κB pathway through the suppression of IKK-mediated phosphorylation and the consequent degradation of IκB, without significantly affecting other TNF-α-activated MAPK kinases, such as JNK, p38, and ERK1/2. Structure–activity analysis has also highlighted the importance of the α-methylene-γ-lactone group, present exclusively in plumericin, for the effective inhibition of the NF-κB pathway, indicating that this chemical structure is a key element of the compound’s anti-inflammatory activity [25].

IS is a ligand and AhR activator, a ligand-activated transcription factor belonging to the Per–Arnt–Sim superfamily of proteins [31]. To further investigate the mechanisms underlying the effect of plumericin in mitigating IS intestinal damage, AhR activation was evaluated by confocal microscopy. We confirmed that IS strongly activates AhR, promoting its expression in IEC-6 treated cells, while plumericin significantly inhibits AhR expression. Activation of AhR by IS is of crucial importance in intestinal homeostasis impairment leading to the generation of ROS through both NADPH oxidase and mitochondrial pathways, thus establishing a pro-oxidant environment that activates NF-κB. At the same time, NF-κB activation contributes to NLRP3 inflammasome priming and activation, further exacerbating epithelial injury and barrier dysfunction [38].

The effect of IS is further evident under conditions of chronic inflammation, such as those simulated by exposure to LPS and IFN [39]. In this study, we recreated a condition involving more severe intestinal inflammation by combining IS with LPS and IFN, being similar to that observed in advanced CKD with inflammatory complications. As previously reported, IS further enhances parameters of inflammation, oxidative stress, and of wound repair mechanisms in IEC-6 in inflammatory conditions [12,16]. The results obtained indicated that plumericin exerts a protective effect also in this more pronounced condition of IEC impairment. In fact, plumericin reduces pro-inflammatory factors such as iNOS and COX-2 and ROS release in IEC-6. Moreover, we also report plumericin’s ability to promote epithelial restoration in wound healing and to increase the expression of E-cadherin and ZO-1, whose distribution is dysregulated by IS in an even more pronounced way in inflammatory conditions. These results emphasize the importance of modulating IS-induced inflammation, especially when it is exacerbated by stimuli such as LPS and IFN.

Plumericin’s capacity to modulate both AhR and NF-κB pathways highlights the potential crosstalk between these routes in CKD-related complications, suggesting that plumericin may interfere with IS-induced signaling at multiple points. Activation of AhR by IS produces ROS through both NADPH oxidase and mitochondrial pathways, creating a pro-oxidant environment that activates NF-κB. Simultaneously, NF-κB activation contributes to NLRP3 inflammasome priming and activation, further aggravating epithelial damage and wound repair mechanisms. Conversely, by reducing AhR expression, plumericin could limit the initial surge of ROS and the subsequent oxidative stress, thereby preventing the activation of both NF-κB and the NLRP3 inflammasome. Furthermore, also the relationship between NF-κB and Nrf2 is complex and bidirectional. The promoter of the NFE2L2 gene, which encodes Nrf2, contains binding sites for both NF-κB and the AhR receptor, as well as xenobiotic response elements (XREs). This establishes Nrf2 as a pivotal node in the regulatory axis connecting oxidative stress, inflammation and uremic toxins [46,48].

Although direct targets of plumericin in the AhR and Nrf2 pathways have not yet been identified, it is possible to hypothesize that the modulation of these factors may be a related effect of NF-κB suppression, contributing to the reduction of oxidative stress and inflammation. although their direct regulation by plumericin has not yet been demonstrated. Future studies will therefore be essential to determine whether the AhR and Nrf2 pathways are modulated independently or as part of a coordinated response, as well as to clarify the temporal sequence of molecular events.

The close interplay between these pathways suggests that this axis could be a promising therapeutic target for limiting the damage caused by uremic toxins in CKD. The convergence between AhR activation, NFκB-dependent inflammation, and Nrf2 impairment is emerging as a critical checkpoint in CKD-associated intestinal dysfunction. Our data suggest that plumericin selectively acts on this triad: it attenuates upstream sensing (AhR), blocks inflammatory amplification (NFκB), and re-establishes antioxidant balance (Nrf2). This integrated mechanism may represent the key distinguishing element of the compound and highlight its potential to counteract IS induced damage at intestinal level in CKD.

## 4. Materials and Methods

### 4.1. Reagents

All compounds and reagents were purchased from Sigma Chemicals Company (Sigma, Milan, Italy), unless stated otherwise.

### 4.2. Plumericin

Plumericin was isolated from the bark of *H. sucuuba*. Detailed description of the phytochemical work, including the isolation and identification of plumericin and other compounds from *H. sucuuba,* was previously provided elsewhere [28]. Details about the storage, stability, and purity of the plumericin were previously described [49].

### 4.3. Cell Culture

The IEC-6 cell line (CRL-1592) was purchased from the American Type Culture Collection (ATCC, Manassas, VA, USA. IEC-6 cells were cultured with Dulbecco’s modified Eagle’s medium (DMEM, 4 g/L glucose) containing 2 mM L-glutamine, 10% (*v*/*v*) fetal bovine serum (FBS), 0.1 unit/mL bovine insulin, and 1.5 g/L NaHCO_3_. For the experiments, IEC-6 cells were used between the 16th–19th passages.

### 4.4. Cellular Treatment

The IEC-6 cells were plated and, after adhesion (24 h), were treated with plumericin (2-1-0-5 μM) for 1 h and then co-exposed to IS (250 μM) alone or to IS + LPS (lipopolysaccharides from *E. coli*; serotype O111:B4; 10 µg/mL) plus interferon-γ (IFN; 10 U/mL) for different experimental times, as highlighted below. The plumericin concentration was established considering previous studies [25,28,29]. In this study we considered a concentration in line with IS accumulation in CKD patients and in line with previous experimental studies [10,28,29,50,51],

#### 4.4.1. Apoptosis Evaluation

The anti-apoptotic activity of plumericin was analyzed by evaluating the percentage of hypodiploid DNA, using propidium iodide (PI) staining by flow cytometry [29]. Briefly, IEC-6 cells (3.5 × 10^5^; 24-well plates) were plated and allowed to adhere. Thereafter, cells were exposed to plumericin (2-1-0.5 μM) for 1 h and then co-exposed to IS (250 μM) for further 24 h. Following treatment, culture medium was removed, cells washed once with PBS, and then resuspended in 500 µL of a solution containing 0.1% (*w*/*v*) sodium citrate and 50 µg/mL PI. Culture medium and PBS were centrifuged, and cell pellets were pooled with cell suspension to retain both dead and living cells for analysis. After incubation at 4 °C for 30 min in the dark, cell nuclei were analyzed with a Becton Dickinson FACScan flow cytometer (FACSscan; Becton Dickinson, Milan, Italy) using the Cell Quest program (version 4; Becton Dickinson, Milan, Italy).

#### 4.4.2. Interleukin 1β Determination

Interleukin 1β (IL-1β) levels were assessed with an Enzyme-Linked Immuno Sorbent Assay (ELISA). IEC-6 cells (2 × 10^4^ cells/well; 96-well plates) were plated, allowed to adhere for 24 h, and then treated as previously indicated. Supernatants from IEC-6 cells were then collected and a commercial kit (Rat IL-1 beta ELISA Kit; Invitrogen BMS630, Waltham, MA, USA) was used to perform the ELISA assay, according to the manufacturer’s instructions (Invitrogen). Results were expressed as pg/mL, as previously reported [28].

#### 4.4.3. Measurement of Cyclooxygenase-2 (COX-2), Inducible Nitric Oxide Synthase (iNOS), Superoxide Dismutase (SOD), and Caspase-1, Bax, Bcl-2, E-Cadherin, ZO-1 and Aryl Hydrocarbon Receptor (AhR) Expression by Cytofluorimetry

IEC-6 cells were plated into 96-well plates (2.0 × 10^3^ cells/ well) and treated as previously indicated, for 24 h. Cells were then collected and washed with phosphate-buffered saline (PBS), followed by the addition of fixing solution (PBS containing 1% BSA, 1% formaldehyde). After 20 min, IEC-6 cells were incubated in a permeabilization buffer (fixing buffer containing 0.1% TritonX) for further 30 min. Anti-cyclooxygenase-2 (COX-2; sc-376861Santa Cruz Biotechnologies, Dallas, TX, USA), anti-inducible nitric oxide synthase (iNOS; sc-7271Santa Cruz Biotechnologies, Dallas, TX, USA), anti-superoxide dismutase (SOD; sc-137254 Santa Cruz Biotechnologies, Dallas, TX, USA), anti-caspase-1 (sc-292736 Santa Cruz Biotechnologies, Dallas, TX, USA), anti-Bax (sc-23959Santa Cruz Biotechnologies, Dallas, TX, USA), anti-Bcl-2 (sc-7282Santa Cruz Biotechnologies, Dallas, TX, USA), anti-E-cadherin (sc-8426Santa Cruz Biotechnologies, Dallas, TX, USA), anti-ZO-1 (594-21773 Proteintech, Rosemont, IL, USA or anti-AhR (B300-515Novus Biologicals, Centennial, CO, USA) antibodies were then added for 30 min with the secondary FITC-conjugated antibody. Cell fluorescence was then evaluated by a fluorescence-activated cell sorter (FACSscan; Becton Dickinson; Milan, Italy) and analyzed by Cell Quest software, as formerly reported (version 4; Becton Dickinson; Milan, Italy) [28].

#### 4.4.4. Intracellular ROS Release Measurement

ROS intracellular release in IEC-6 cells was evaluated using the probe 2′,7′-dichlorofluorescin-diacetate (H_2_DCF-DA). Cells (2.0 × 10^3^ cells/well) were plated into 96-well plates and treated as previously indicated, for 24 h. Cells were then washed with PBS and incubated in HBSS containing H_2_DCF-DA (10 µM) at 37 °C for 30 min. Thereafter, cell fluorescence was evaluated using the EnSpire Multimode Plate Reader (PerkinElmer, Milan, Italy).

#### 4.4.5. Wound-Healing Assay

In order to evaluate cellular migration in IS-treated IEC-6 cells, a wound-healing assay was performed. IEC-6 cells (1 × 10^5^ cells/well, 24-well plates) were allowed to adhere for 24 h. A mechanical wound was induced at the center of the IEC-6 monolayer by scraping cells with a sterile plastic p200 pipette tip. Cells were then washed with PBS and treated as previously indicated. A 5× phase contrast objective (Axiovert 5, Zeiss, Jena, Germany) was used to record cell movements immediately after treatment (time 0) and at the end of the assay (24 h). Statistical analyses were performed using GraphPad Prism 8 software (GraphPad, San Diego, CA, USA).

#### 4.4.6. Immunofluorescence Analysis for NF-κB, the Nuclear Factor Erythroid Derived 2 (Nrf2), AhR, and ZO-1 by Confocal Microscopy

IEC-6 cells were plated on coverslips in a 24-well plate (8.0 × 10^4^ cells /well) and allowed to adhere for 24 h. Plumericin (1μM) was then added to the cells for 1 h alone and after IS (250 μM) or IS + LPS + IFN was added for 1 h to evaluate p65-NF-κB subunit, Nrf2 nuclear translocation, and ZO-1 localization. Cells were then fixed with 4% paraformaldehyde in PBS and permeabilized with 0.1% Triton in PBS, and the blocking was performed with bovine serum albumin (BSA) and PBS. Cells were then incubated with mouse anti-p65 NF-κB antibody (sc-8008SantaCruz Biotechnologies), rabbit anti-Nrf2 antibody (A34499Novus Biologicals), or AhR antibody (Novusbiologicals NB300-515) for 1 h at 37 °C; for ZO-1 detection, cells were incubated overnight with anti-ZO-1 antibody (594-21773 Santa Cruz Biotechnologies). Subsequently, PBS was used to wash the slides, and the fluorescein-conjugated (FITC) secondary antibody was added for 1 h. 4,6-Diamidine-2-O-phenylindole dihydrochloride (DAPI) was used for the nuclei identification, as formerly reported [16]. Finally, the coverslips were mounted in mounting medium and images were acquired using the laser confocal microscope (Leica TCS SP5, Leica, Wetzlar, Germany). Fluorescence intensity quantification was performed using ImageJ software (version 1.54g; National Institutes of Health, Bethesda, MD, USA. Regions of interest (ROIs) were defined for each image, and the average signal intensity value was measured.

#### 4.4.7. Data Analysis

Data are reported as mean ± standard deviation of the mean (SD) of at least three independent experiments conducted at least in triplicate. The Shapiro–Wilk test was performed to verify data normality and the variance test (ANOVA) followed by Bonferroni’s test was applied by using GraphPad Prism 8 (GraphPad software). A *p*-value of less than 0.05 was considered significant.

## 5. Conclusions

In conclusion, despite future studies being necessary to better elucidate the interplay between the possible integrating mechanism/s underlying plumericin activity, this study reports the protective effect of plumericin on IS-induced intestinal damage, and thus plumericin interest in the specific context of intestinal impairment in CKD, reinforcing the importance of in-depth mechanistic studies to fully understand its pharmacological profile.

## Data Availability

Dataset available on request from the authors.

## Supplementary Materials

The following supporting information can be downloaded at: https://www.mdpi.com/article/10.3390/ijms27010293/s1.

## Abbreviations

The following abbreviations are used in this manuscript:

AhR	aryl hydrocarbon receptor
CKD	chronic kidney disease
COX-2	cyclooxygenase-2
DAPI	4,6-diamidine-2-O-phenylindole dihydrochloride
DCF	2′,7′-dichlorofluorescein
ELISA	enzyme-linked immuno sorbent assay
GSH	L-glutathione reduced
HO-1	heme oxygenase-1
H_2_DCF-DA	2′,7′-dichlorofluorescin-diacetate
IFN-γ	interferon-γ
iNOS	inducible nitric oxide synthase
IS	indoxyl sulfate
L-name	N(ω)-nitro- l-arginine methyl ester
LPS	lipopolysaccharides
NOX	NADPH oxidase
NF-kB	nuclear factor-kB
NQO1	NAD(P)H dehydrogenase quinone 1
Nrf2	nuclear factor (erythroid-derived 2)-like 2
PBS	phosphate buffer saline
PI	propidium iodide
ROS	reactive oxygen species
SOD	superoxide dismutase
TNF-α	tumor necrosis factor-α
