# Supplementary material for: Plumericin Modulates the AhR–NFκB–Nrf2 Signaling Network to Counteract Indoxyl Sulfate-Induced Intestinal Epithelial Cells Impairment
Source: Int J Mol Sci. 2025 Dec 27;27(1):293. doi: 10.3390/ijms27010293 (PMC12785376; doi:10.3390/ijms27010293)
Supplement: Supplementary file 1 [file ijms-27-00293-s001.zip › ijms-4058749-supplementary.pptx]

## Slide 1
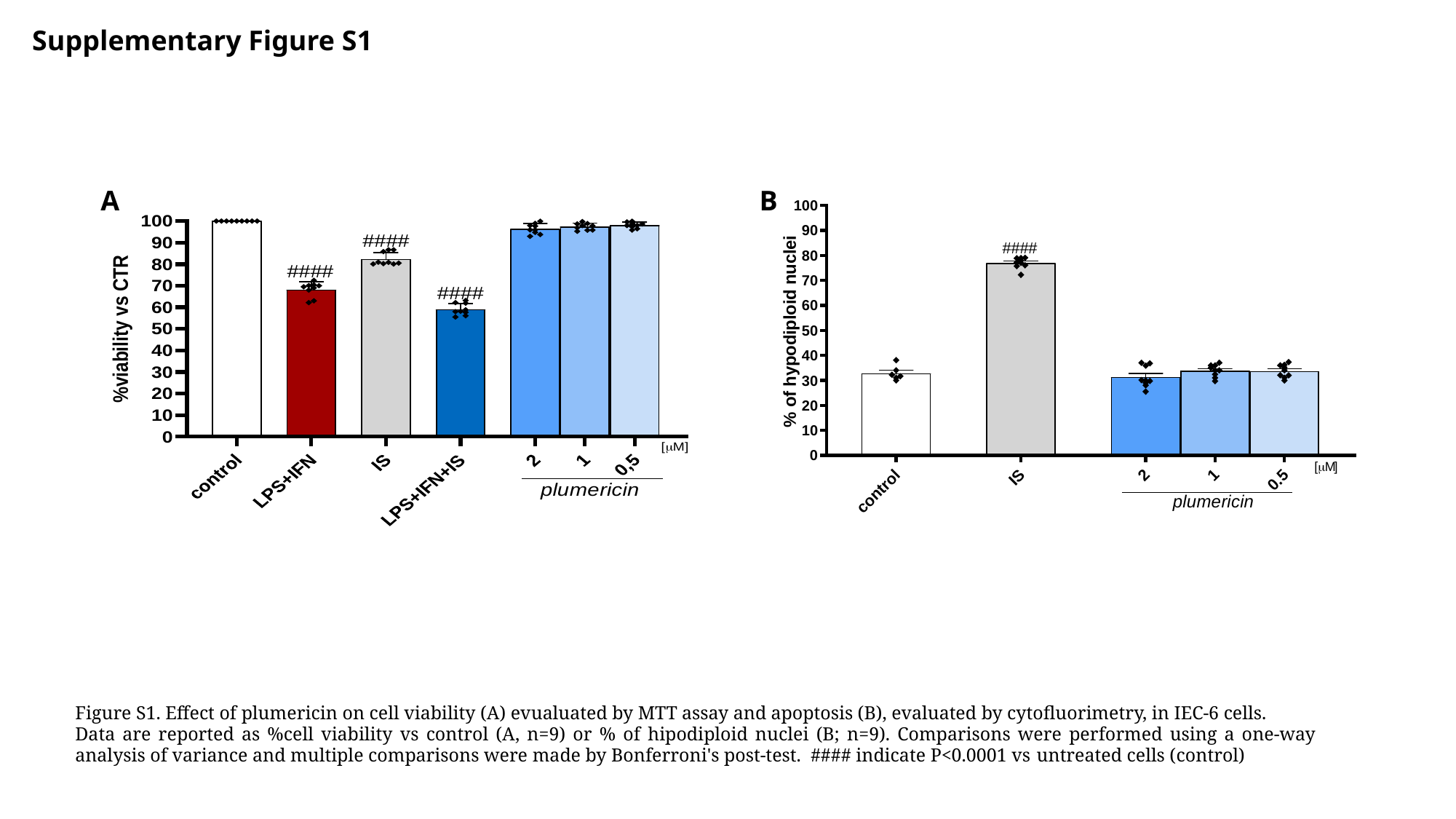

Supplementary Figure S1
A
B
Figure S1. Effect of plumericin on cell viability (A) evualuated by MTT assay and apoptosis (B), evaluated by cytofluorimetry, in IEC-6 cells.
Data are reported as %cell viability vs control (A, n=9) or % of hipodiploid nuclei (B; n=9). Comparisons were performed using a one-way analysis of variance and multiple comparisons were made by Bonferroni's post-test. #### indicate P<0.0001 vs untreated cells (control)

## Slide 2
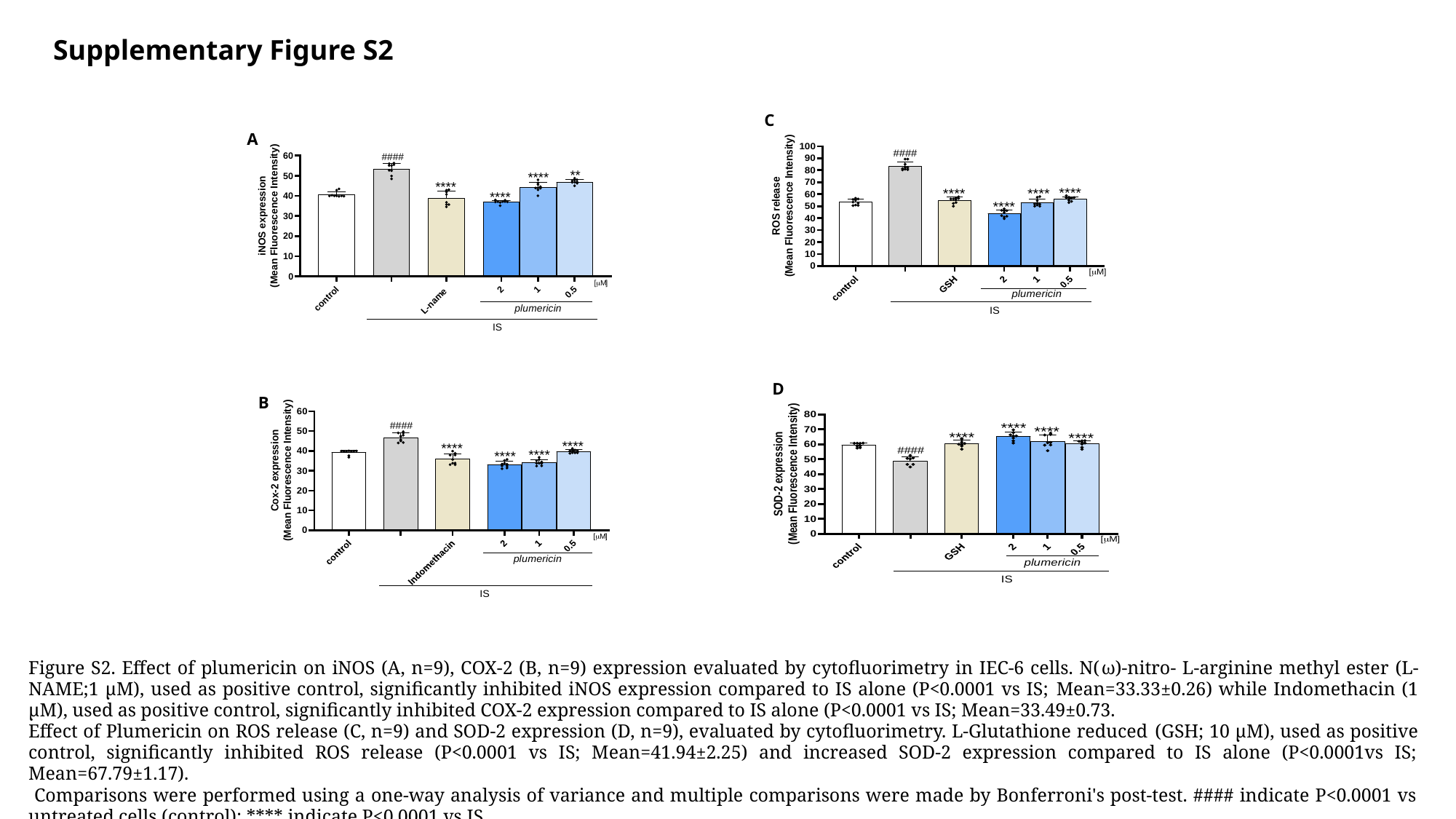

Supplementary Figure S2
C
A
D
B
Figure S2. Effect of plumericin on iNOS (A, n=9), COX-2 (B, n=9) expression evaluated by cytofluorimetry in IEC-6 cells. N(ω)-nitro- l-arginine methyl ester (L-NAME;1 μM), used as positive control, significantly inhibited iNOS expression compared to IS alone (P<0.0001 vs IS; Mean=33.33±0.26) while Indomethacin (1 μM), used as positive control, significantly inhibited COX-2 expression compared to IS alone (P<0.0001 vs IS; Mean=33.49±0.73.
Effect of Plumericin on ROS release (C, n=9) and SOD-2 expression (D, n=9), evaluated by cytofluorimetry. L-Glutathione reduced (GSH; 10 µM), used as positive control, significantly inhibited ROS release (P<0.0001 vs IS; Mean=41.94±2.25) and increased SOD-2 expression compared to IS alone (P<0.0001vs IS; Mean=67.79±1.17).
 Comparisons were performed using a one-way analysis of variance and multiple comparisons were made by Bonferroni's post-test. #### indicate P<0.0001 vs untreated cells (control); **** indicate P<0.0001 vs IS.

## Slide 3
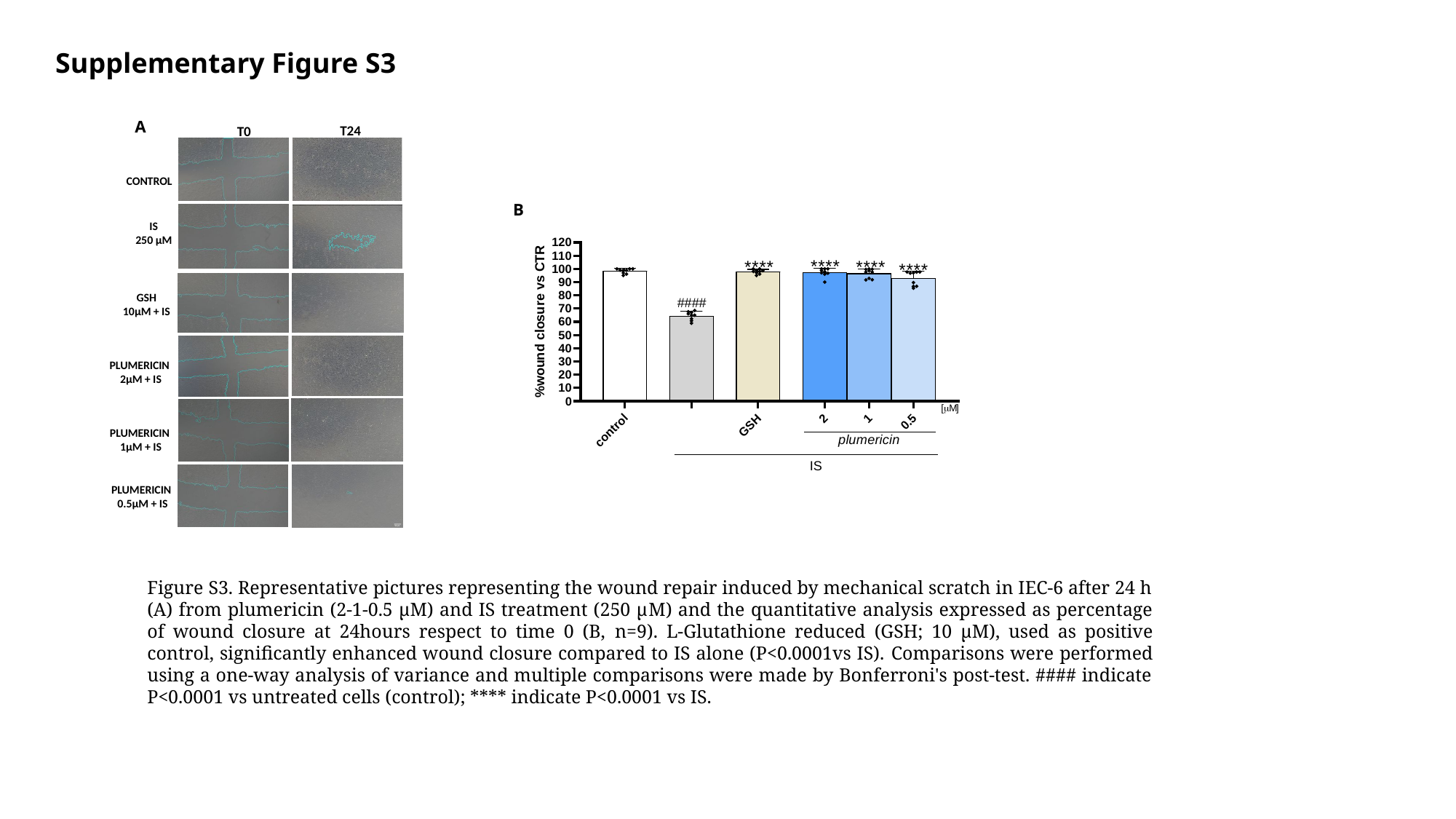

Supplementary Figure S3
A
T24
T0
CONTROL
B
IS
250 μM
GSH
10μM + IS
PLUMERICIN
2μM + IS
PLUMERICIN
1μM + IS
PLUMERICIN
0.5μM + IS
Figure S3. Representative pictures representing the wound repair induced by mechanical scratch in IEC-6 after 24 h (A) from plumericin (2-1-0.5 µM) and IS treatment (250 μM) and the quantitative analysis expressed as percentage of wound closure at 24hours respect to time 0 (B, n=9). L-Glutathione reduced (GSH; 10 µM), used as positive control, significantly enhanced wound closure compared to IS alone (P<0.0001vs IS). Comparisons were performed using a one-way analysis of variance and multiple comparisons were made by Bonferroni's post-test. #### indicate P<0.0001 vs untreated cells (control); **** indicate P<0.0001 vs IS.

## Slide 4
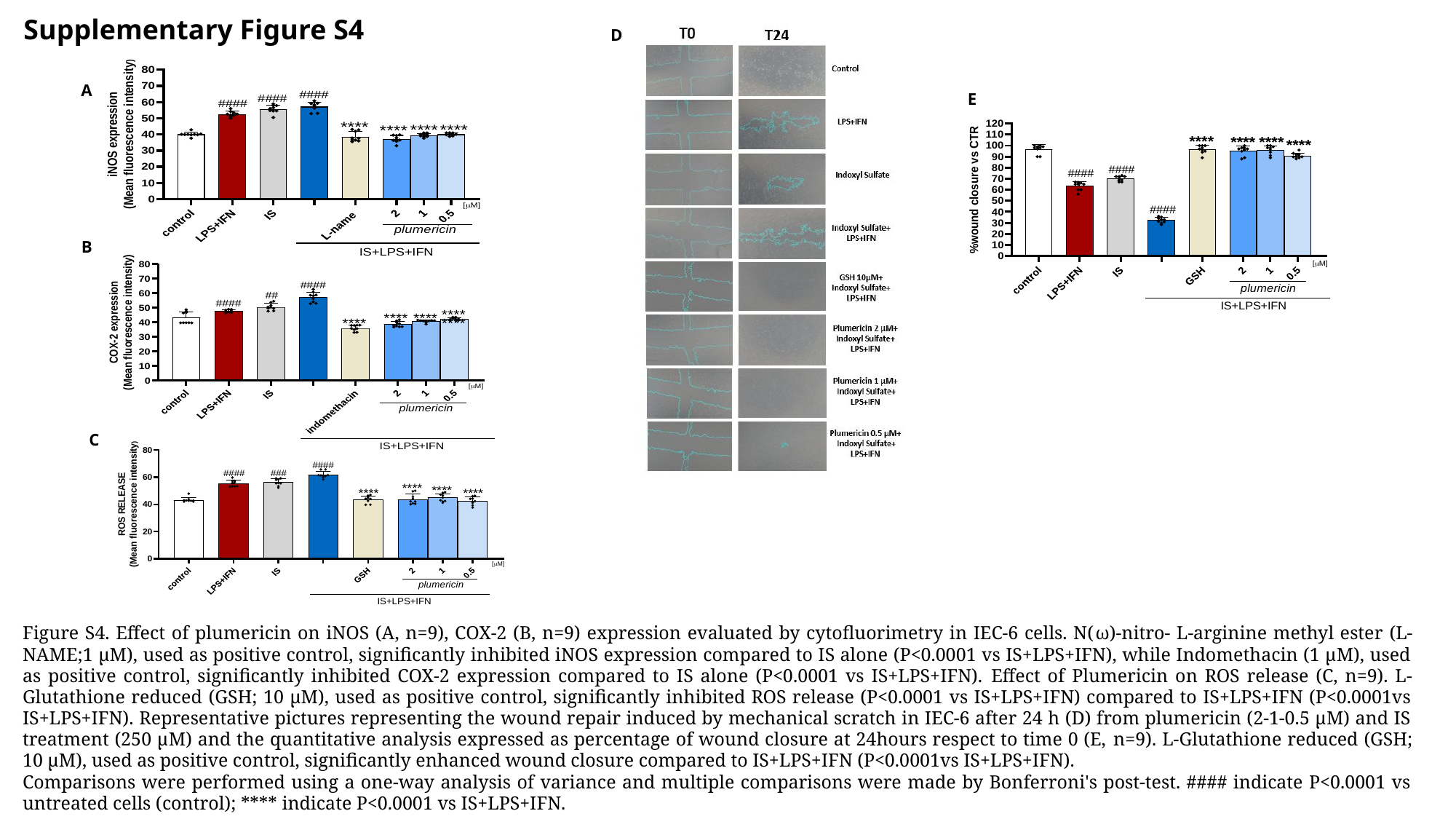

Supplementary Figure S4
D
A
E
B
C
Figure S4. Effect of plumericin on iNOS (A, n=9), COX-2 (B, n=9) expression evaluated by cytofluorimetry in IEC-6 cells. N(ω)-nitro- l-arginine methyl ester (L-NAME;1 μM), used as positive control, significantly inhibited iNOS expression compared to IS alone (P<0.0001 vs IS+LPS+IFN), while Indomethacin (1 μM), used as positive control, significantly inhibited COX-2 expression compared to IS alone (P<0.0001 vs IS+LPS+IFN). Effect of Plumericin on ROS release (C, n=9). L-Glutathione reduced (GSH; 10 µM), used as positive control, significantly inhibited ROS release (P<0.0001 vs IS+LPS+IFN) compared to IS+LPS+IFN (P<0.0001vs IS+LPS+IFN). Representative pictures representing the wound repair induced by mechanical scratch in IEC-6 after 24 h (D) from plumericin (2-1-0.5 µM) and IS treatment (250 μM) and the quantitative analysis expressed as percentage of wound closure at 24hours respect to time 0 (E, n=9). L-Glutathione reduced (GSH; 10 µM), used as positive control, significantly enhanced wound closure compared to IS+LPS+IFN (P<0.0001vs IS+LPS+IFN).
Comparisons were performed using a one-way analysis of variance and multiple comparisons were made by Bonferroni's post-test. #### indicate P<0.0001 vs untreated cells (control); **** indicate P<0.0001 vs IS+LPS+IFN.
